# 4,4′-[Oxalylbis(azanediyl)]dipyridinium bis(perchlorate)

**DOI:** 10.1107/S1600536810041760

**Published:** 2010-10-20

**Authors:** Wayne Hsu, Hui-Lin Hsiao, Jhy-Der Chen

**Affiliations:** a Department of Chemistry, Chung-Yuan Christian University, Chung-Li, Taiwan

## Abstract

In the title molecular salt, C_12_H_12_N_4_O_2_^2+^·2ClO_4_^−^, the complete cation is generated by crystallographic inversion symmetry. In the crystal, the cations and anions are linked *via* N—H⋯O and N—H⋯(O,O) hydrogen bonds, forming a three-dimensional framework.

## Related literature

For the applications of *N*,*N*′-bis­(pyrid­yl)oxamides, see: Hsu *et al.* (2004 ▶); Hu *et al.* (2004 ▶).
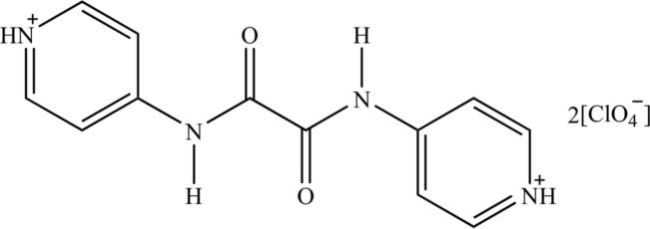


## Experimental

### 

#### Crystal data

C_12_H_12_N_4_O_2_^2+^·2ClO_4_^−^
*M* *_r_* = 443.16
Monoclinic, 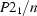

*a* = 7.873 (1) Å
*b* = 9.3728 (15) Å
*c* = 11.3205 (16) Å
β = 94.827 (10)°
*V* = 832.4 (2) Å^3^
*Z* = 2
Mo *K*α radiation
μ = 0.46 mm^−1^
*T* = 295 K
0.6 × 0.4 × 0.2 mm

#### Data collection

Bruker P4 diffractometer
Absorption correction: ψ scan (*XSCANS*; Siemens, 1995 ▶) *T*_min_ = 0.919, *T*_max_ = 0.982
2017 measured reflections
1450 independent reflections
921 reflections with *I* > 2σ(*I*)
*R*_int_ = 0.038
3 standard reflections every 97 reflections  intensity decay: none

#### Refinement

*R*[*F* ^2^ > 2σ(*F* ^2^)] = 0.051
*wR*(*F* ^2^) = 0.119
*S* = 1.03
1450 reflections
127 parameters
H-atom parameters constrained
Δρ_max_ = 0.31 e Å^−3^
Δρ_min_ = −0.30 e Å^−3^

### 

Data collection: *XSCANS* (Siemens, 1995 ▶); cell refinement: *XSCANS*; data reduction: *SHELXTL* (Sheldrick, 2008 ▶); program(s) used to solve structure: *SHELXS97* (Sheldrick, 2008 ▶); program(s) used to refine structure: *SHELXL97* (Sheldrick, 2008 ▶); molecular graphics: *SHELXTL*; software used to prepare material for publication: *SHELXTL*.

## Supplementary Material

Crystal structure: contains datablocks I, global. DOI: 10.1107/S1600536810041760/gk2310sup1.cif

Structure factors: contains datablocks I. DOI: 10.1107/S1600536810041760/gk2310Isup2.hkl

Additional supplementary materials:  crystallographic information; 3D view; checkCIF report

## Figures and Tables

**Table 1:** Hydrogen-bond geometry (Å, °)

*D*—H⋯*A*	*D*—H	H⋯*A*	*D*⋯*A*	*D*—H⋯*A*
N1—H1*A*⋯O4	0.86	2.21	2.950 (4)	144
N1—H1*A*⋯O3^i^	0.86	2.35	2.966 (5)	129
N2—H2*A*⋯O2^ii^	0.86	2.14	2.975 (5)	162

Symmetry codes: (i) 
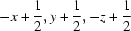
; (ii) 
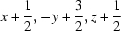
.

**Fig. 1.**
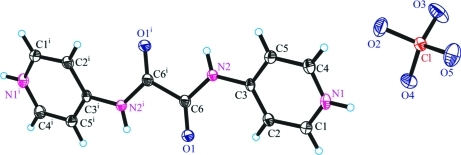
Crystal structure of the title compound with atom labeling and displacement ellipsoids drawn at the 30% probability level. Symmetry code: i =-x + 1,-y + 2,-z + 2.

## supplementary crystallographic information

### Comment

Several Ag(I) complexes containg *N*,*N'*-bis(2-pyridyl)oxamide ligands have been prepared, which show one-dimensional and two-dimensional structures (Hsu, *et al.*, 2004; Hu, *et al.*, 2004). To investigate the effect of ligand-isomerism on the structural type of such complexes, the ligand *N*,*N'*-bis(4-pyridyl)oxamide was synthesized and reacted with AgClO_4_ in CH_2_Cl_2_. The reaction resulted unexpectedly in the perchlorate salt of the organic ligand. Within this project the crystal structure of the title compound was determined.

### Experimental

*N*,*N'*-bis(4-pyridyl)oxamide (0.24 g, 1.0 mmol) and AgClO_4_ (0.21 g, 1.0 mmol) were placed in a flask containing 10 ml CH_2_Cl_2_. The mixture was then reflux for 12 h. The resulting solution was then filtered and then layered with diethyl ether to afford coloress plate crystals of the title compound suitable for X-ray crystallography.

### Refinement

All the hydrogen atoms were placed in idealized positions and refined using the riding model approximation with *C*—H = 0.93 — 0.96 Å, N—H = 0.86 Å and *U*_iso_(H) = 1.2 *U*_eq_(C, N).

### Crystal data

**Table:**

C_12_H_12_N_4_O_2_^2+^·2ClO_4_^−^	*F*(000) = 452
*M**_r_* = 443.16	*D*_x_ = 1.768 Mg m^−^^3^
Monoclinic, *P*2_1_/*n*	Mo *K*α radiation, λ = 0.71073 Å
Hall symbol: -P 2yn	Cell parameters from 25 reflections
*a* = 7.873 (1) Å	θ = 5.6–14.2°
*b* = 9.3728 (15) Å	µ = 0.46 mm^−^^1^
*c* = 11.3205 (16) Å	*T* = 295 K
β = 94.827 (10)°	Plate, colorless
*V* = 832.4 (2) Å^3^	0.6 × 0.4 × 0.2 mm
*Z* = 2	

### Data collection

**Table:**

Bruker P4 diffractometer	921 reflections with *I* > 2σ(*I*)
Radiation source: fine-focus sealed tube	*R*_int_ = 0.038
graphite	θ_max_ = 25.0°, θ_min_ = 2.8°
ω scans	*h* = −9→1
Absorption correction: ψ scan (*XSCANS*; Siemens, 1995)	*k* = −1→11
*T*_min_ = 0.919, *T*_max_ = 0.982	*l* = −13→13
2017 measured reflections	3 standard reflections every 97 reflections
1450 independent reflections	intensity decay: none

### Refinement

**Table:**

Refinement on *F*^2^	Primary atom site location: structure-invariant direct methods
Least-squares matrix: full	Secondary atom site location: difference Fourier map
*R*[*F*^2^ > 2σ(*F*^2^)] = 0.051	Hydrogen site location: inferred from neighbouring sites
*wR*(*F*^2^) = 0.119	H-atom parameters constrained
*S* = 1.03	*w* = 1/[σ^2^(*F*_o_^2^) + (0.0424*P*)^2^ + 0.6192*P*] where *P* = (*F*_o_^2^ + 2*F*_c_^2^)/3
1450 reflections	(Δ/σ)_max_ < 0.001
127 parameters	Δρ_max_ = 0.31 e Å^−^^3^
0 restraints	Δρ_min_ = −0.30 e Å^−^^3^

### Special details

**Table:**

Experimental. Refinement of *F*^2^ against ALL reflections. The weighted *R*-factor *wR* and goodness of fit *S* are based on *F*^2^, conventional *R*-factors *R* are based on *F*, with *F* set to zero for negative *F*^2^. The threshold expression of *F*^2^ > σ(*F*^2^) is used only for calculating *R*-factors(gt) *etc*. and is not relevant to the choice of reflections for refinement. *R*-factors based on *F*^2^ are statistically about twice as large as those based on *F*, and *R*- factors based on ALL data will be even larger.
Geometry. All e.s.d.'s (except the e.s.d. in the dihedral angle between two l.s. planes) are estimated using the full covariance matrix. The cell e.s.d.'s are taken into account individually in the estimation of e.s.d.'s in distances, angles and torsion angles; correlations between e.s.d.'s in cell parameters are only used when they are defined by crystal symmetry. An approximate (isotropic) treatment of cell e.s.d.'s is used for estimating e.s.d.'s involving l.s. planes.
Refinement. Refinement of *F*^2^ against ALL reflections. The weighted *R*-factor *wR* and goodness of fit *S* are based on *F*^2^, conventional *R*-factors *R* are based on *F*, with *F* set to zero for negative *F*^2^. The threshold expression of *F*^2^ > σ(*F*^2^) is used only for calculating *R*-factors(gt) *etc*. and is not relevant to the choice of reflections for refinement. *R*-factors based on *F*^2^ are statistically about twice as large as those based on *F*, and *R*- factors based on ALL data will be even larger.

### Fractional atomic coordinates and isotropic or equivalent isotropic displacement parameters (Å^2^)

**Table:**

	*x*	*y*	*z*	*U*_iso_*/*U*_eq_	
C1	0.3752 (6)	1.1939 (5)	0.5833 (3)	0.0381 (11)	
H1B	0.3929	1.2868	0.5582	0.046*	
C2	0.4314 (5)	1.1532 (5)	0.6960 (3)	0.0326 (10)	
H2C	0.4865	1.2182	0.7482	0.039*	
C3	0.4053 (5)	1.0143 (4)	0.7310 (3)	0.0248 (9)	
C4	0.2689 (6)	0.9652 (5)	0.5391 (3)	0.0382 (11)	
H4B	0.2127	0.9029	0.4851	0.046*	
C5	0.3260 (5)	0.9182 (5)	0.6505 (3)	0.0327 (10)	
H5A	0.3118	0.8234	0.6718	0.039*	
C6	0.4772 (5)	1.0472 (4)	0.9445 (3)	0.0288 (9)	
N1	0.2943 (5)	1.0996 (4)	0.5092 (3)	0.0376 (9)	
H1A	0.2575	1.1276	0.4394	0.045*	
N2	0.4560 (4)	0.9647 (4)	0.8451 (2)	0.0285 (8)	
H2A	0.4754	0.8748	0.8533	0.034*	
O1	0.4640 (4)	1.1740 (3)	0.9511 (2)	0.0426 (8)	
Cl	0.07220 (14)	0.92622 (11)	0.21648 (8)	0.0338 (3)	
O2	0.0153 (5)	0.8460 (4)	0.3141 (2)	0.0579 (10)	
O3	0.1889 (4)	0.8433 (4)	0.1564 (3)	0.0653 (11)	
O4	0.1543 (4)	1.0522 (3)	0.2623 (2)	0.0539 (9)	
O5	−0.0718 (4)	0.9610 (4)	0.1369 (3)	0.0598 (10)	

### Atomic displacement parameters (Å^2^)

**Table:**

	*U*^11^	*U*^22^	*U*^33^	*U*^12^	*U*^13^	*U*^23^
C1	0.057 (3)	0.031 (2)	0.027 (2)	0.002 (2)	0.005 (2)	0.0014 (19)
C2	0.040 (3)	0.034 (3)	0.0221 (18)	0.000 (2)	−0.0045 (19)	−0.0037 (18)
C3	0.027 (2)	0.031 (2)	0.0155 (18)	0.000 (2)	−0.0032 (16)	0.0006 (17)
C4	0.047 (3)	0.041 (3)	0.025 (2)	0.000 (2)	−0.0038 (19)	−0.003 (2)
C5	0.040 (2)	0.031 (2)	0.0267 (19)	0.001 (2)	−0.0010 (18)	0.001 (2)
C6	0.032 (2)	0.032 (3)	0.0213 (19)	0.001 (2)	−0.0044 (17)	−0.0023 (19)
N1	0.047 (2)	0.046 (2)	0.0185 (15)	0.002 (2)	−0.0052 (15)	0.0025 (17)
N2	0.039 (2)	0.0258 (19)	0.0195 (16)	0.0027 (16)	−0.0035 (14)	−0.0014 (14)
O1	0.071 (2)	0.0297 (18)	0.0259 (15)	0.0076 (17)	−0.0039 (14)	−0.0017 (13)
Cl	0.0438 (6)	0.0314 (6)	0.0252 (5)	0.0015 (6)	−0.0035 (4)	−0.0029 (5)
O2	0.087 (3)	0.051 (2)	0.0344 (15)	−0.015 (2)	−0.0001 (17)	0.0134 (16)
O3	0.061 (2)	0.077 (3)	0.058 (2)	0.019 (2)	0.0025 (18)	−0.034 (2)
O4	0.082 (2)	0.035 (2)	0.0436 (17)	−0.0117 (18)	0.0001 (17)	−0.0096 (15)
O5	0.0471 (19)	0.080 (3)	0.0489 (18)	0.005 (2)	−0.0158 (16)	0.0135 (19)

### Geometric parameters (Å, °)

**Table:**

C1—N1	1.342 (5)	C5—H5A	0.9300
C1—C2	1.368 (5)	C6—O1	1.196 (5)
C1—H1B	0.9300	C6—N2	1.363 (4)
C2—C3	1.382 (6)	C6—C6^i^	1.555 (7)
C2—H2C	0.9300	N1—H1A	0.8600
C3—C5	1.391 (5)	N2—H2A	0.8600
C3—N2	1.399 (4)	Cl—O3	1.419 (3)
C4—N1	1.324 (5)	Cl—O4	1.423 (3)
C4—C5	1.375 (5)	Cl—O5	1.425 (3)
C4—H4B	0.9300	Cl—O2	1.439 (3)
			
N1—C1—C2	119.9 (4)	O1—C6—N2	127.6 (4)
N1—C1—H1B	120.0	O1—C6—C6^i^	122.0 (4)
C2—C1—H1B	120.0	N2—C6—C6^i^	110.4 (4)
C1—C2—C3	119.1 (4)	C4—N1—C1	122.7 (3)
C1—C2—H2C	120.5	C4—N1—H1A	118.6
C3—C2—H2C	120.5	C1—N1—H1A	118.6
C2—C3—C5	119.4 (3)	C6—N2—C3	125.3 (3)
C2—C3—N2	122.7 (3)	C6—N2—H2A	117.3
C5—C3—N2	117.9 (4)	C3—N2—H2A	117.3
N1—C4—C5	119.6 (4)	O3—Cl—O4	109.7 (2)
N1—C4—H4B	120.2	O3—Cl—O5	109.6 (2)
C5—C4—H4B	120.2	O4—Cl—O5	110.7 (2)
C4—C5—C3	119.2 (4)	O3—Cl—O2	109.7 (2)
C4—C5—H5A	120.4	O4—Cl—O2	108.32 (18)
C3—C5—H5A	120.4	O5—Cl—O2	108.8 (2)

Symmetry codes: (i) −*x*+1, −*y*+2, −*z*+2.

### Hydrogen-bond geometry (Å, °)

**Table:**

*D*—H···*A*	*D*—H	H···*A*	*D*···*A*	*D*—H···*A*
N1—H1A···O4	0.86	2.21	2.950 (4)	144
N1—H1A···O3^ii^	0.86	2.35	2.966 (5)	129
N2—H2A···O2^iii^	0.86	2.14	2.975 (5)	162

Symmetry codes: (ii) −*x*+1/2, *y*+1/2, −*z*+1/2; (iii) *x*+1/2, −*y*+3/2, *z*+1/2.
